# Supplementary material for: Our Healthy Clarence: A Community-Driven Wellbeing Initiative
Source: Int J Environ Res Public Health. 2019 Sep 30;16(19):3691. doi: 10.3390/ijerph16193691 (PMC6801779; doi:10.3390/ijerph16193691)

Supplementary Data—Assessment of media reporting

Supplementary methods

Media articles were sourced via a search of the ProQuest Australia & New Zealand Newsstream database. The search strategy encompassed terms to capture reporting related to suicide within the Clarence Valley and its main towns of Grafton, Yamba, Maclean (for the major river) and the Our Healthy Clarence initiative itself: (Our Healthy Clarence) OR (suicide AND (Grafton OR Yamba OR MacLean OR Clarence Valley)) time limited to the years 2015-2018.

Titles and abstracts were screened for relevance to the search goals – articles were included if they were about the Clarence Valley and about suicide, suicide prevention and/or Our Healthy Clarence. Articles relating to euthanasia, court cases or non-local were excluded.

Articles were assessed and scored for message tone, with a scoring guide of +1 for positive messaging or strengths/solutions focussed reporting, 0 for neutral or ambiguous reporting, and -1 for crisis and/or problem focussed reporting. Where uncertainty lay, the article was read in full. These were then plotted against the date of publication (see below).

Supplementary results –

The search yielded 1,822 results including duplicate articles. After screening for relevance, 239 articles were included and assessed for tone as described as above. These scores were plotted against date of publication and this is represented in Supplementary Figure S1 below.

Figure S1 – Tone of media reporting with respect to suicide, suicide prevention and Our Healthy Clarence (2015-2018)

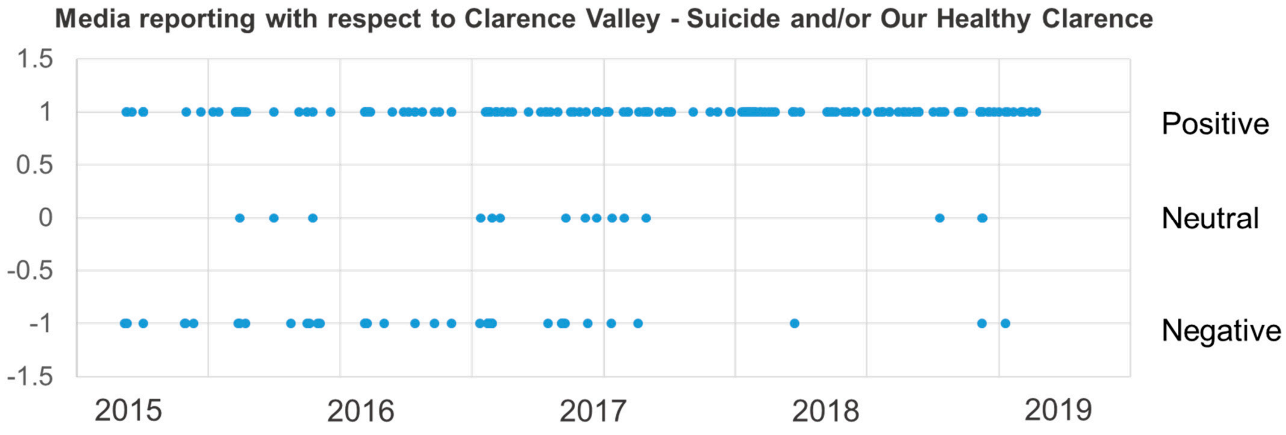

Supplement: Supplementary file 1 [file ijerph-16-03691-s001.pdf]
